# Expecting the Unexpected: Infants Use Others’ Surprise to Revise Their Own Expectations

**DOI:** 10.1162/opmi_a_00117

**Published:** 2024-03-01

**Authors:** Yang Wu, Megan Merrick, Hyowon Gweon

**Affiliations:** Department of Psychology, University of Toronto Scarborough, Toronto, ON, Canada; Department of Psychological and Brain Sciences, Indiana University Bloomington, Bloomington, IN, USA; Department of Psychology, Stanford University, Stanford, CA, USA

**Keywords:** statistical reasoning, vicarious prediction error, emotion understanding, social learning, looking time

## Abstract

Human infants show systematic responses to events that violate their expectations. Can they also revise these expectations based on others’ expressions of surprise? Here we ask whether infants (*N* = 156, mean = 15.2 months, range: 12.0–18.0 months) can use an experimenter’s expression of surprise to revise their own expectations about statistically probable vs. improbable events. An experimenter sampled a ball from a box of red and white balls and briefly displayed either a surprised or an unsurprised expression at the outcome before revealing it to the infant. Following an unsurprised expression, the results were consistent with prior work; infants looked longer at a statistically improbable outcome than a probable outcome. Following a surprised expression, however, this standard pattern disappeared or was even reversed. These results suggest that even before infants can observe the unexpected events themselves, they can use others’ surprise to *expect the unexpected*. Starting early in life, human learners can leverage social information that signals others’ prediction error to update their own predictions.

## INTRODUCTION

Prediction error is a central notion in theories of learning across domains, including neuroscience, cognitive science, and machine learning (Daw & Doya, 2006; Gershman & Niv, 2010; Schultz et al., 1997). The general idea is that the discrepancy between an expected outcome and the actual outcome can serve as information for learning. Consistent with this idea, developmental research has found an early-emerging sensitivity to unexpected (i.e., surprising) outcomes: When infants observe an event that is inconsistent with their expectations, they show a range of behavioral (Aslin, 2007; Bonawitz et al., 2012; Sim & Xu, 2017, 2019; Stahl & Feigenson, 2015; Theobald & Brod, 2021), neural (Berger et al., 2006; Kouider et al., 2015; Wilcox et al., 2005), and physiological (Jackson & Sirois, 2009) changes. Most notably, infants look longer (Aslin, 2007; Sim & Xu, 2019) when an event violates their expectations than when it does not.

To benefit from one’s own prediction error, learners must use their prior knowledge to generate an expectation, and critically, observe the event that violates their expectation. For instance, given a box that contains mostly red and just a few white balls, even infants look longer when a random sample from the box turns out to be white, rather than red (Xu & Garcia, 2008). Despite the extensive body of research that relies on infants’ responses to their *own* prediction error, relatively little is known about whether infants can also leverage *others’* prediction error to inform their own inferences. That is, what if infants see someone peeking at the outcome and looking surprised, before they have a chance to see the outcome themselves? If infants can interpret this person’s expression as an indication that something unexpected has happened, they might update their own prediction, too; it is probably white. Such an inference can be characterized as using someone else’s surprise to “expect the unexpected.”

Prior research has demonstrated that humans draw rich inferences from information provided by others starting early in life (Gweon, 2021). Yet, the developmental origins of our ability to leverage others’ prediction error as a source of information remains poorly understood. When another person’s response to an unknown event signals their prediction error, can infants use this to update their own inferences, even before directly observing the event themselves? Synthesizing prior work on infants’ expectations about physical events (Spelke, 2022) and their ability to leverage others’ emotional expressions to inform their own inferences (Wu et al., 2021), here we ask whether infants can use others’ expression of surprise—an indicator of others’ prediction error—to revise their own expectations about the world.

A critical cognitive prerequisite for making such an inference is the ability to interpret the meaning of others’ responses to events, such as their emotional expressions. Research suggests that sensitivity to others’ emotional expressions emerges early in life. Within the first year of life, infants can differentiate positive emotional expressions from negative ones, and match facial and vocal expressions cross-modally (Grossmann, 2010; Ruba & Repacholi, 2020). By 12 months, infants begin to interpret others’ emotional expressions as conveying information about the world (Wu et al., 2021). For instance, infants can use the valence of another person’s emotional expression to decide whether to approach or avoid an ambiguous situation (Clément & Dukes, 2017; Walle et al., 2017). Furthermore, they can use others’ emotional responses to events to infer what others were attending to (Moll et al., 2006, 2007) and modulate their own exploration accordingly (Wu et al., 2017). Taken together, these findings raise the possibility that infants can interpret others’ expressions of surprise as an indication that something unexpected has happened, and use it to update their own expectations.

To address this possibility, we adapted a looking-time task from prior work on infants’ sensitivity to statistically probable vs. improbable events (Denison et al., 2013; Denison & Xu, 2019; Xu & Garcia, 2008). While infants’ sensitivity to others’ surprise may manifest in a wide range of events (e.g., physical and psychological events), we chose statistical events as an example domain; this is because prior work has shown that infants can not only make inferences about a sampling outcome based on the population (Denison et al., 2013; Denison & Xu, 2019; Xu & Garcia, 2008) but also incorporate multiple sources of information into their inferences, such as physical constraints (Denison & Xu, 2010a; Téglás et al., 2007) and psychological states of the sampling agent (Gweon et al., 2010; Xu & Denison, 2009). Thus, statistical sampling provides a well-established context for asking whether infants can also incorporate others’ emotional expressions into their inferences. More specifically, we hypothesize that the effect of someone else’s surprise on infants’ inferences can manifest as a reduction, or even a reversal, of the well-established pattern of longer looking at improbable events.

Our task was similar to the sampling events used in Xu and Garcia (2008), which provided initial evidence for infants’ inferences about statistically improbable events. In that study, infants saw an experimenter randomly draw five balls from a box that contained a biased proportion of red and white balls (e.g., mostly-red or mostly-white); infants looked longer at a statistically improbable outcome (i.e., 1 red and 4 white balls from a mostly-red box) than a probable outcome (i.e., the same sample from a mostly-white box).

**Figure 1.**
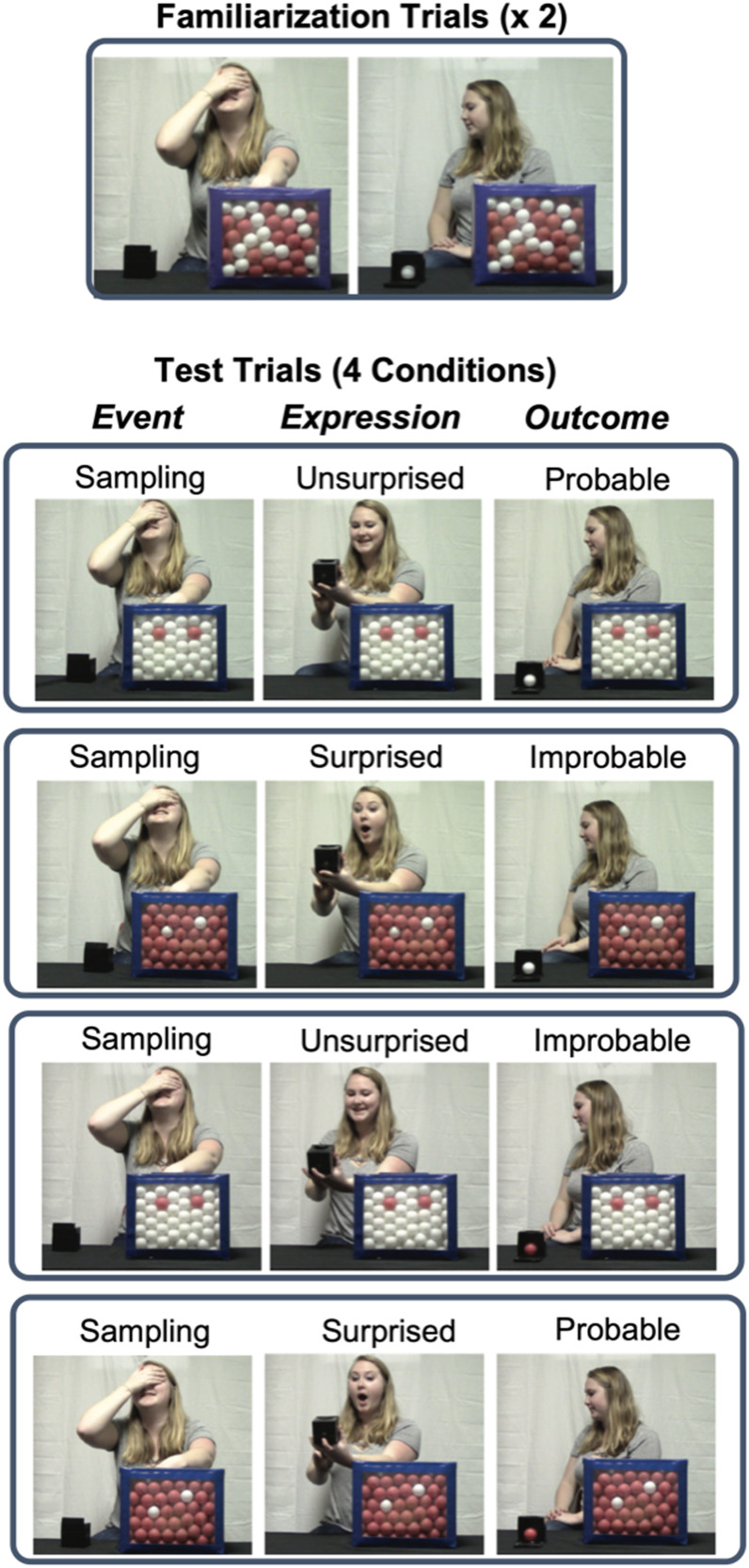
Design and procedure of Experiments 1 and 2 (see Footnote 2 for the link to demo videos). In Experiment 1, the two emotional expressions alternated across trials (Unsurprised first, as shown in the figure, or Surprised first). In Experiment 2a, two Surprised trials always preceded two Unsurprised trials; in Experiment 2b, two Unsurprised trials always preceded two Surprised trials. The color of balls was counterbalanced in all experiments.

To investigate the effect of others’ surprise, we made two critical changes to the original task. First, the experimenter drew just one ball instead of five. Although this change makes our improbable outcome relatively less improbable compared to prior work,^1^ it was critical for constraining the number of possible outcomes to only two (i.e., either a red ball or a white ball, rather than a broad range of possibilities) such that revising an initial expectation (e.g., a red ball) meant expecting the other outcome (i.e., a white ball). Second, before revealing the sampling outcome to infants, the experimenter “peeked” at the outcome and displayed either a surprised expression or an unsurprised (mildly positive) expression (see Figure 1). We varied both the probability of the outcome (Probable vs. Improbable) and the experimenter’s emotional expression (Surprised vs. Unsurprised) across four trials (within-subject).

We hypothesized that the effect of outcome probability would be modulated by the presence or absence of the experimenter’s surprise. Thus, our primary prediction was an interaction between the probability of outcome and the experimenter’s emotional expression. More specifically, we predicted that when the experimenter’s expression does not indicate surprise, the pattern of results would mirror prior findings (i.e., longer looking to the improbable outcome than to the probable outcome; see Denison et al., 2013; Denison & Xu, 2019; Xu & Garcia, 2008). However, if infants can use others’ surprise to modulate their own expectations, they might find an improbable outcome relatively *less* surprising; thus, the standard pattern of looking time would disappear (i.e., similar looking to the probable and improbable outcome), or even be reversed (i.e., longer looking to the probable outcome than to the improbable outcome).

Although the ability to predict the sample given the population in random sampling events may be present well before 12 months of age (e.g., Denison et al., 2013; Xu & Denison, 2009; Xu & Garcia, 2008), it remains unclear when infants begin to modulate these inferences based on others’ emotional responses. Given prior work suggesting that infants between 12 and 17 months old can infer the probable causes of others’ emotional expressions (Moll et al., 2006, 2007; Wu et al., 2017), we recruited infants in the same age range for our study.

Given the design of our study, we anticipated the effect in our key condition—longer looking at probable than improbable outcomes in the Surprised Trials—to be rather small. This is because the sampling event used in our task induces a baseline tendency in the opposite direction (i.e., longer looking at improbable than probable outcomes), which we assume to be preserved when the experimenter expresses no surprise before revealing the outcome (the Unsurprised trials). In the Surprised trials, if infants can use others’ surprise to revise their expectations, this would manifest as a reduction or reversal of the standard pattern of looking time only if infants can override this baseline tendency.

Additionally, our design involved sequential presentation of trials that varied in both outcome probability and emotional expression. While this within-subject design yields more data from each participant and reduce inter-subject variability, it also means that infants would see the experimenter respond to the same outcomes in two very different ways across trials, one of which was particularly salient and attention-grabbing (i.e., surprise). This raises the possibility that the order of trials might affect the results. Accordingly, we first report an initial experiment using a counterbalanced emotion order (Experiment 1, *N* = 28), followed by two preregistered experiments using particular emotion orders (Experiment 2a, Surprised Trials First, *N* = 64; Experiment 2b, Unsurprised Trials First; *N* = 64).

## EXPERIMENT 1

### Method

#### Participants.

For this initial investigation, we did not have clear grounds for estimating the effect size for power analysis. Thus, we chose a sample size—*N* = 28—which was used in our conceptual replication of Xu and Garcia (2008; see SOM) and is larger than most of the infant studies in literature (Oakes, 2017). To maximize usable data, we first applied a priori exclusion criteria at the trial level, and excluded a participant if more than half (two) of the test trials were unusable. The final sample included *N* = 28 infants recruited from a local museum (mean: 15.5 months, range: 12.9–18.0; 10 girls, 18 boys), with full data from 19 infants and partial data from 9 infants. Partial data resulted from the exclusion of 12 trials due to fussiness (5 trials), parental/sibling interference (2 trials), and experimenter error (5 trials). Another four infants had insufficient data after trial-level exclusion and were excluded from further analyses. Additionally, a researcher had mistakenly turned on a noisy fan in the testing room for four consecutive testing days; all data from these sessions (12 infants) were excluded.

#### Materials.

We made two boxes (30 cm × 24 cm × 30 cm) with carton board. One box was used for familiarization trials and the other for test trials. The front and back sides of the boxes were transparent. Following Xu and Garcia (2008), we divided the inside of each box into three “secret” compartments (front, middle, back) and filled each compartment with a mix of red and white ping pong balls. While the experimenter always reached into the middle compartment through an opening on top of the box, to a naïve observer, it appeared as though she drew balls from the entire box. For the familiarization box, both the front and back compartments were filled with 50% white and 50% red ping-pong balls and the box appeared to contain half red and half white balls when seen from both sides. For the test box, the front compartment was filled with 95% white and 5% red ping-pong balls and the reverse for the back compartment. Presenting the stimuli using two opposite sides of a single box allowed us to switch the proportion of balls presented to participants quickly and conveniently.

To present the sampling outcome, we used a container that was already pre-loaded with either a white or a red ball, so that the experimenter could pretend to draw a ball from the box and place it into the container through an opening on top. A total of six containers (2 for familiarization, 4 for main trials) were created for this purpose. The front side could be opened to reveal its content, but the covering had two opaque layers. Thus, the experimenter could open just the first layer to appear as if she “peeked” at the outcome and express either surprise or no surprise; to reveal the content to the infant, she could face the opening towards the infant and open both layers. Finally, to make the sampling event as realistic as possible, a small speaker was hidden inside the container which played a pre-recorded ball-dropping sound just as the experimenter pretended to drop the sampled ball inside. We presented videos of the sampling event to three naïve research assistants and asked them to describe what the experimenter just did. All of them believed that the experimenter actually drew a ball from the big box and placed it inside the container. Using this box-to-container sampling setup allowed us to (1) control the sampling outcome, (2) ensure that infants could not see the outcome until the experimenter revealed it to them, and (3) give the impression that the experimenter “peeked” at the outcome while keeping the experimenter masked to condition throughout the whole session.

#### Procedure.

Each infant was tested in a private room inside a local museum. The infant sat on their parent’s lap, approximately 1.5 meters away from the experimenter. The experimenter greeted the infant and said: “I’m going to show you a box!”, which marked the beginning of the study.

Infants first saw two familiarization trials designed to introduce the random sampling procedure (see Figure 1 and Movie S1^2^). In each trial, the experimenter took out a big box covered by a piece of black felt and a small opaque container. She removed the felt from the box and said: “Look!” The box appeared to contain 50% red and 50% white ping-pong balls based on its transparent front. The experimenter shook the box and looked at the front of the box to indicate that she saw the distribution of balls. Then she said: “I’m going to take a ball! Look at me!” She covered her eyes with one hand and reached into the box with the other hand, giving the impression that she is randomly drawing a ball from the box. She then said: “I got one, and I put it in here!”, and appeared to take a ball out of the box and drop it into the container; in reality, she was merely pretending to draw a ball, and the container was already pre-loaded with a ball. Then she opened her eyes and revealed the ball inside the container to the infant, saying: “Look what I got!” The container had a red ball in one familiarization trial and a white ball in the other (order counterbalanced). Then she looked away with a neutral expression for five seconds.

Then infants saw four test trials (see Figure 1 and Movies S2–S5) which were similar to the familiarization trials with two exceptions. First, the big box either contained 95% red balls and 5% white balls or vice versa, such that a ball in the majority color would be a more likely sample (i.e., expected) than a ball in the minority color (i.e., unexpected). Second, after the experimenter dropped the sampled ball into the container, she turned the front side of the container towards herself and opened its covering, revealing its content to herself but not to the infant; she then made either a surprised expression (Surprised Condition) or a mildly happy, unsurprised expression (Unsurprised Condition) while looking at the content of the container. In reality, she opened only the first layer of the container’s cover and was therefore unable to see the color of the ball. This ensured that the experimenter was masked to condition. While maintaining her expression, the experimenter then looked at the infant for a second (to keep the infant engaged and to make sure that the infant saw the expression) and then looked back at the container. After this, the experimenter returned to her baseline facial expression (neutral to slightly positive) and revealed the ball to the infant by opening both layers of the front cover, saying, “Look what I got!”

The experimenter alternated her emotional expression (i.e., surprised or unsurprised) every trial; whether she showed surprise or no surprise in the first trial was counterbalanced across participants. The experimenter also alternated the color of the majority ball in the box every trial, and the majority color in the first trial was counterbalanced across participants. The color of the sampled ball was the same in the first two trials, making one outcome probable and the other improbable; the other color was used in the last two trials, also making one outcome probable and the other improbable, but the order of outcome probability was reversed. In other words, the order of outcome probability across trials was either probable-improbable-improbable-probable or improbable-probable-probable-improbable; this order allowed for counterbalancing outcome probability within participants.

At the end of each trial, the experimenter looked away with a completely neutral expression (see Figure 1). While looking away, she monitored the infant’s looking responses from a hidden laptop connected to a webcam. She also secretly recorded the infant’s looking behavior by pressing a button on a hidden mouse when the infant was looking at the stage and releasing the button when the infant looked away. A MatLab script was used to determine when the infant had looked away from the stage for two consecutive seconds. When the 2-second criterion was reached, it was programmed to emit a subtle auditory signal for the experimenter to proceed to the next trial.

The experimenter’s judgments served primarily as a conservative cutoff to determine when to proceed to the next trial. All looking time data used for analyses were offline coded independently by a primary coder (also masked to condition); the primary coder reviewed the video recordings from all experimental sessions and coded the period from the moment the outcome was revealed to the participant until the participant looked away from the stage for a continuous period of two seconds. When the primary coder judged that the experimenter ended a trial prematurely, the trial was excluded (4 trials, categorized as cases of “experimenter error” in data exclusion; see Participants). To minimize any bias, a second offline coder, also masked to condition, independently coded all looking responses from videotape in the same way as the primary coder. Inter-coder reliability between the two offline coders was *r* = .97.

### Results

**Figure 2.**
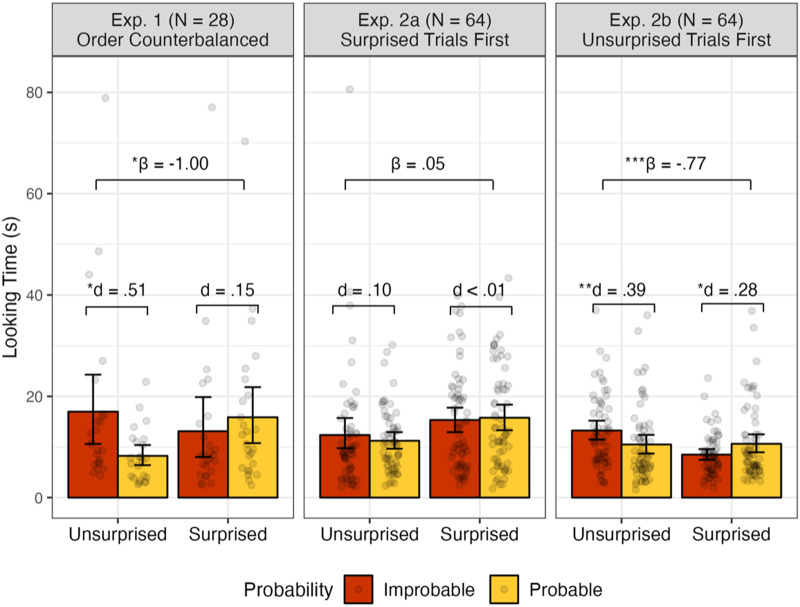
Results of Experiment 1 (initial study) and Experiment 2 (preregistered study). Raw looking time in seconds, with error bars indicating 95% confidence intervals. The standardized *β* coefficient (*β*) indicates the effect size of the interaction effect between Probability and Emotion, while Cohen’s *d* (*d*) indicates the effect size of pairwise comparisons. Significance levels are denoted by the symbols “*,” “**,” and “***,” representing *p* < .05, *p* < .01, and *p* < .001, respectively. Both effect sizes and significance levels were calculated based on log-transformed data due to their non-normal (right-skewed) distribution; see Results for details.

As the raw looking time data were right-skewed (see Figure 2) and violated the assumption of normality (*p* < 10^−13^; the Shapiro-Wilk test of normality, *p* > .05 suggests a normal distribution), we followed the recommendation to log-transform the data before excluding outliers and analysis (Csibra et al., 2016). The data showed near-normal distribution after log transformation (*p* = .043) and all data points were within three deviations of the mean.

We analyzed the log-transformed data with a linear mixed-effects model (using the *nlme* package in *R*; Pinheiro et al., 2021) in which Probability, Emotion, and the Probability-by-Emotion interaction were fixed effects. We also included a maximal random effect structure such that random intercepts and random slopes of Probability, Emotion, and their interaction were all fit by subject.^3^

As predicted, there was an interaction between Probability and Emotion (b coefficent (*B*) = −.77, standardized *β* coefficient (*β*) = −1.00, *t*(69) = −2.59, *p* = .012). See Figure 2. Infants looked longer when the outcome was improbable than when it was probable (*t*(19) = 2.30, *p* = .033, 95% CI [.04, .84], Cohen’s *d* = .51; paired *t* test). This effect was absent in the Surprised trials (*t*(23) = −.74, *p* = .468, 95% CI [−.54, .26], Cohen’s *d* = .15). See Figure 2. These results suggest that the tendency to look longer at the improbable sampling outcome (Xu & Garcia, 2008) was relatively larger in the Unsurprised trials than in the Surprised trials.

To further explore the effect of trial order, we analyzed each trial separately with a focus on the first trial, which couldn’t have been affected by the experimenter’s emotional expressions in the preceding trials. Note however that this analysis included only about 6 to 7 infants in each trial type, and was thus severely under-powered. The pattern of the first trial data mirrored the overall pattern. There was an interaction between Probability and Emotion (*B* = −1.45, *β* = −1.83, *t* = −2.40, *p* = .026). Infants looked longer at an improbable than a probable outcome in the Unsurprised trial (*t*(4.96) = 3.07, *p* = .028, 95% CI [.19, 2.22], Cohen’s *d* = 2.17; welch two sample *t* test). This effect was absent in the Surprised trial (*t*(11.86) = −.59, *p* = .565, 95% CI [−1.12, .64], Cohen’s *d* = .30). No effect was found in the remaining trials (*p*s > .193 for all pairwise comparisons).

Together, the results from Experiment 1 were overall consistent with our predictions. Yet, as anticipated, the effect in the Surprised condition was rather small, and our analyses may have been under-powered due to the small sample size.

## EXPERIMENT 2

In Experiment 2, we sought to find clearer and stronger evidence for our hypothesis. We made the following changes to our sample size and design.

First, given the pattern in the first-trial data in Experiment 1, we recruited a sample large enough for analyzing just the first-trial data from each participant in order to test our hypothesis without concerns about possible contamination from preceding trials. To ensure sufficient power, we tested 64 participants who saw a Surprised expression on the first trial (Experiment 2a) and 64 participants who saw an Unsurprised expression on the first trial (Experiment 2b).

Second, we modified our design to better understand how the order of emotional expressions might influence infants’ looking time. Rather than alternating emotional expressions across four trials as in Experiment 1, we first presented two trials that use one emotional expression, followed by two trials that use the other expression: In Experiment 2a, infants always saw two Surprised trials followed by two Unsurprised trials, whereas in Experiment 2b, infants always saw two Unsurprised trials followed by two Surprised trials (outcome probability counterbalanced within each pair). This design allowed us to explore the effect of seeing surprised expressions earlier vs. later in the course of four trials. All other aspects of Experiments 2a and 2b were the same as Experiment 1, including the age of infants and the order of outcome probability.

We did not have strong a priori predictions about which experiment would yield more robust effects: The effect of surprise might be stronger in earlier trials (hence stronger effects in Experiment 2a), or seeing more sampling events without surprise might amplify the effect of surprise in later trials (hence stronger effects in Experiment 2b). However, given that concurrently running both experiments with a large sample size would require a considerable amount of time, we deliberately prioritized Experiment 2a which presents the Surprised trials first. The rationale was that if the first trials in Experiment 2a show significantly longer looking time at the probable than the improbable outcome, this would provide the clearest evidence that the experimenter’s surprise can “reverse” the effect of outcome probability, without any potential influence from preceding trials. However, given that Experiment 1 was under-powered and did not provide conclusive evidence for such a reversal, we anticipated that we might run Experiment 2b to collect data in both orders. Experiment 2b was preregistered and conducted following the completion of Experiment 2a, and the preregistration included analyses that combine both datasets^4^. We report both experiments together below.

### Method

#### Participants.

As in Experiment 1, we recruited infants from a local museum. In addition to the trial-level and participant-level exclusion criteria used in Experiment 1, we also preregistered that we would exclude participants whose first-trial data were excluded or missing. The final sample for Experiment 2a included *N* = 64 infants (mean: 14.9 months, range: 12.2–17.9; 29 girls, 35 boys), with full data from 54 infants and partial data from 10 infants. Partial data resulted from the exclusion of 11 trials due to fussiness (3 trials), parental/sibling interference (2 trials), or experimenter error (6 trials). An additional 17 infants had insufficient data after trial-level exclusion and were excluded from further analyses.

Our final sample for Experiment 2b included *N* = 64 infants (mean: 15.5 months, range: 12.0–17.9; 31 girls, 33 boys), with full data from 56 infants and partial data from 8 infants. Partial data resulted from the exclusion of 10 trials due to fussiness (2 trials), parental/sibling interference (3 trials), distraction (2 trials), experimenter error (2 trials), and data outside three standard deviations (1 trial). An additional 11 infants had insufficient data after trial-level exclusion and were excluded from further analyses.

#### Materials.

We used the same materials as in Experiment 1.

#### Procedure.

The procedure was identical to Experiment 1, but instead of fully counterbalancing the two emotional expressions across trials, we manipulated the order of emotional expressions. In Experiment 2a, the experimenter always expressed surprise in the first two trials and expressed no surprise in the following two trials. Experiment 2b used the reverse order: The experimenter always expressed no surprise in the first two trials and expressed surprise in the following two trials. We used the same procedure to code infants’ looking time as in Experiment 1. Across both experiments, only one trial (in Experiment 2b) was excluded due to premature termination by the experimenter (categorized as a case of “experimenter error” in data exclusion; see Participants). Inter-coder reliability between the primary and second offline coders was *r* = .98 (Experiment 2a) and *r* = .95 (Experiment 2b).

### Results

The raw looking time data violated the assumption of normality in both Experiments 2a and 2b (both *p*s < 10^−12^, the Shapiro-Wilk test of normality; see Figure 2). While our preregistration did not explicitly state whether log-transformation would be applied, due to the skewed distributions, we followed the recommendation to log-transform the data before exclusion and analyses as in Experiment 1 (Csibra et al., 2016). The resulting data were normally distributed (*p* = .086 in Experiment 2a and *p* = .484 in Experiment 2b). Following preregistration, datapoints outside three standard deviations of the mean were excluded from further analyses.

Examining just the first trial of Experiments 2a and 2b, we found a pattern of results that is consistent with Experiment 1. Our preregistered confirmatory analysis focused on the comparison between infants’ looking time at improbable and probable outcomes in the first trial of each experiment. We found that infants looked longer at an Improbable than a Probable outcome when the experimenter was not surprised (i.e., the first trial of Experiment 2b; *t*(58.77) = 2.27, *p* = .027, 95% CI [.04, .69], Cohen’s *d* = .57; Welch two-sample *t*-test), and this effect disappeared when the experimenter was surprised (i.e., the first trial of Experiment 2a; *t*(61.88) = −1.03, *p* = .309, 95% CI [−.55, .18], Cohen’s *d* = .26). Combining the first-trial data from both Experiments 2a and 2b, we found that the interaction between Emotion and Outcome Probability was also significant (*B* = −.56, *β* = −.78, *t* = −2.28, *p* = .025).^5^ These results mirror the pattern of results in Experiment 1, providing additional support for our hypothesis.

Analyzing Experiments 2a and 2b separately but including all four trials in each experiment (a pre-registered exploratory analysis), we found different results depending on the order of emotional expressions.

In Experiment 2a, where the Surprised trials were presented before the Unsurprised trials, no effects were observed. There was no interaction between Probability and Emotion (*B* = .04, *β* = .05, *t*(178) = .22, *p* = .824) and infants did not distinguish between the Improbable and Probable outcomes in either the Unsurprised trials (*t*(53) = −.75, *p* = .455, 95% CI [−.33, .15], Cohen’s *d* = .10; paired *t* test) or the Surprised trials (*t*(62) = −.04, *p* = .965, 95% CI [−.22, .21], Cohen’s *d* < .01). See Figure 2. These results suggest that seeing the experimenter express surprise—a salient, attention-grabbing expression—early in the study might have disrupted infants’ inferences in later Unsurprised trials, making even the well-established effect of event probability (i.e., longer looking to improbable than probable outcomes) disappear.^6^

In Experiment 2b, however, where the Surprised trials were presented after the Unsurprised trials, we found not only an effect in the Unsurprised trials but also a reversal of this effect in the Surprised trails. Specifically, there was an interaction between Probability and Emotion (*B* = −.46, *β* = −.77, *t*(179) = −3.63, *p* < .001). While infants looked longer at an Improbable than a Probable outcome following an unsurprised expression (*t*(58) = 2.97, *p* = .004, 95% CI [.09, .48], Cohen’s *d* = .39; paired *t* test), this pattern was reversed when the experimenter expressed surprise: Infants looked significantly longer at a Probable than an Improbable outcome (*t*(59) = −2.15, *p* = .036, 95% CI [−.33, −.01], Cohen’s *d* = .28). See Figure 2. These results suggest that seeing Unsurprised trials first may have facilitated infants’ inferences in the Surprised trials. Given that only the Surprised trials required infants to revise their expectations, seeing the simpler, Unsurprised trials early in the study might have better prepared infants to modulate their expectations in the Surprised trials that followed.

## GENERAL DISCUSSION

In this study, we found that infants’ looking time to an event outcome is modulated by others’ expressions of surprise. In the absence of surprised expressions, the results were in line with prior findings: Infants (12.0–18.0 months old) looked longer at an improbable than a probable outcome. Following a surprised expression, however, this effect was reduced or even reversed. In both experiments, these effects appeared in the first trial and were modulated by the order of emotional expressions in subsequent trials, with the clearest evidence emerging when the Unsurprised trials preceded the Surprised trials (Experiment 2b). Going beyond prior work on how infants respond to events that violate their own expectations about event outcomes (e.g., Stahl & Feigenson, 2015; Xu & Garcia, 2008), these findings suggest that infants can also use others’ expression of surprise as an indication of others’ prediction error, and revise their own expectations accordingly.

These results are in line with a growing literature suggesting that emotional expressions are an important source of information for human learning and reasoning (Wu et al., 2021). Beyond integrating information such as the physical constraints of a sampling event and the psychological states of other people (Denison & Xu, 2010a, 2010b, 2014; Gweon et al., 2010; Téglás et al., 2007; Xu & Denison, 2009), infants can also integrate others’ surprised expressions into their statistical inferences. Such inferences are likely supported by a developing intuitive theory of how other people’s emotional expressions are generated (Doan et al., 2023). Starting early in life, children begin to understand how an agent’s internal mental states and observed external events give rise to emotional responses (e.g., Asaba et al., 2019; Doan et al., 2018; Harris et al., 1989; Lara et al., 2019; Scott, 2017; Wellman & Bartsch, 1988), and increasingly use observed emotional expressions to make inverse inferences about unobserved events (Egyed et al., 2013; Moll et al., 2006, 2007; Wu & Gweon, 2021; Wu et al., 2017) as well as latent contents of others’ minds (Lagattuta et al., 1997; Repacholi & Gopnik, 1997; Wu et al., 2018; Wu & Schulz, 2018, 2020). While the previous work has mostly focused on inferences based on valenced emotional expressions, our study suggests that even nonvalenced expressions such as surprise support rich inferences early in development.

Although we used sampling events to test our hypotheses, infants’ ability to use others’ surprise to update their inferences may not be constrained to statistically probable or improbable samples. Instead, they might manifest in a broad range of domains insofar as infants have sufficient prior knowledge to generate an initial prediction (e.g., intuitive physics and psychology). For instance, imagine someone who repeatedly chooses a teddy bear over a ball while an observer is present; if this person reaches for one of the two toys again and the observer looks surprised, would infants infer that this person chose a ball instead of a teddy bear? Indeed, infants and young children can use others’ emotional expressions to guide their attention and modulate their exploration in a variety of events (Moll et al., 2006; Tomasello & Haberl, 2003; Wu & Gweon, 2021), suggesting that the inferences found here may not be limited to sampling events. Additionally, the emotional expressions that signal others’ prediction error may not be constrained to surprise alone; depending on the context, other emotions such as disappointment or puzzlement may also imply that someone’s predictions have been violated. The current study represents initial steps towards bridging prior work on individual learning from prediction error (Daw & Doya, 2006; Gershman & Niv, 2010; Schultz et al., 1997; Stahl & Feigenson, 2015), inferential social learning (Gweon, 2021), and emotion understanding (Wu et al., 2021), and opens up directions for further research that integrates the notion of prediction error with inferences in social contexts (Jara-Ettinger, 2019; Vélez & Gweon, 2021).

While our results were overall consistent with our hypotheses, some analyses yielded relatively small effect sizes, and the clearest evidence was found when the Unsurprised trials preceded the Surprised trials (Experiment 2b). Why might this be? First, we suspect that infants’ baseline tendency to look longer at an improbable outcome—which must be overrided in the Surprised trials (but preserved or amplified in the Unsurprised trials)—may have resulted in the relatively small effects in the Surprised trials. Indeed, in each of our experiments, we observed a smaller effect size in the Surprised trials compared to the Unsurprised trials (see Cohen’s *d* values in Results and Figure 2), providing support for this explanation. Further, Experiment 2 suggests that the effects (in both the Unsurprised and Surprised trials) were modulated by the order of emotions. When the Surprised trials preceded the Unsurprised trials (Experiment 2a), the well-established looking-time pattern disappeared even in the Unsurprised trials. This suggests that seeing the experimenter’s surprise early in the study may have been excessively attention-grabbing and salient for infants, weakening the effects in subsequent trials. Additionally, when the Surprised trials followed the Unsurprised trials (Experiment 2b), we not only found longer looking to improbable trials (i.e., baseline effect) in the Unsurprised trials but also a significant effect in the *opposite* direction (i.e., a reversal of the baseline effect) in the Surprised trials. This suggests that seeing the Unsurprised trials early in the study not only allowed infants to show the “standard” pattern in these trials, but also may have facilitated their inferences in later Surprised trials. Given that only the Surprised trials required the infants to revise their expectations, the simpler, Unsurprised trials may have prepared them to better modulate their expectations based on other people’s surprise.

These results also raise important questions about the nature of representations and inferential processes that drive infants’ looking time in our study. Although the results are consistent with our hypothesis that infants used others’ surprise to revise their initial expectations about the outcome, one might wonder if infants only engaged in retrospective reasoning after seeing the final outcome, or were simply responding to the alignment between the revealed outcome and the experimenter’s expression. That is, rather than updating their expectations about the outcome when the experimenter expressed surprise, infants might have found the outcome inconsistent with their memory of the experimenter’s expression only after the final outcome was revealed, and responded to this misalignment. Indeed, prior work shows that infants respond to the mismatch between an event outcome and someone else’s emotional response (Scott, 2017); they expect someone to express surprise when an outcome is unexpected and express satisfaction when the outcome is expected. Similarly, studies using explicit measures found that by age six, children can explicitly reason that the probability of an outcome influences others’ surprise (Doan et al., 2018). In both studies, however, participants saw the event outcome *before* seeing another person’s emotional responses, suggesting that participants’ responses were primarily about the person’s emotional responses (rather than the outcome). In our study, by contrast, participants saw the two events in the reverse order: Infants saw someone’s surprised response to a hidden outcome, which lasted for no more than 2–3 seconds, and then saw the outcome when the surprised expression was no longer available. The alternative account that relies on mere misalignment between past expression and current outcome would suggest that infants were not making any use of the experimenter’s expression when it was actually available, and instead waited until the final outcome was revealed to retrieve their memory of the experimenter’s past emotional expression and assess the misalignment. While the current study cannot conclusively rule out the misalignment account, it poses significant computational demands for retaining and integrating all available information after the outcome is revealed. Prior work suggests that infants can integrate physical and social information to predict sampling outcomes (Denison & Xu, 2010a, 2010b, 2014; Gweon et al., 2010; Téglás et al., 2007; Xu & Denison, 2009), and more recent work has shown that infants can use emotional cues (e.g., positive emotional vocalizations) to draw inferences about, and search accordingly for, the likely causes of those vocalizations (i.e., something adorable or delicious; Wu et al., 2017). In light of these findings, the current results are most consistent with the possibility that infants can also integrate emotional information—the presence or absence of others’ surprise in particular—to make inferences about statistical events.

Yet, one open question concerns the richness of infants’ representations of others’ surprise; did infants in our study consider the experimenter’s initial expectation to interpret her surprise? One possibility is that infants may already have a mentalistic understanding of others’ surprise (Scott, 2017); upon observing the experimenter’s surprise, they may have recognized that the experimenter had an initial expectation of the outcome that was then violated, and use this information to update their own predictions. Alternatively, infants may have simply associated surprised expressions with unexpected outcomes, and directly used those social cues to update their expectations without necessarily representing what the experimenter believed or expected. While some findings suggest that infants and children can interpret others’ emotional expressions based on the emoter’s mental states such as knowledge or beliefs (Moll et al., 2006, 2007; Wu & Gweon, 2021) or even abstract qualities such as reliability (Poulin-Dubois & Brosseau-Liard, 2016; see also Kidd et al., 2013), the current findings are consistent with either possibility.

The current findings also raise new questions for researchers across domains and disciplines. First, our results highlight the importance of studying the sociocultural variability of emotional expressions in young children’s environment. Although the current study recruited infants in the U.S., given the variability in how emotions are expressed across individuals and cultures (Malatesta & Haviland, 1982; Matsumoto, 1990; Tsai, 2007), such variability might also affect how infants from different socio-cultural backgrounds use others’ emotional expressions to inform their own inferences. Second, our results also highlight the educational and clinical significance of emotional expressions. While prior work has shown the effect of early emotional environment on infants’ emotional development (Burris et al., 2019; Pollak, 2008), the current study suggests that it may also affect infants’ cognitive development such as their learning and reasoning about the physical world. Beyond inspiring more research about the downstream consequences of emotional environment on early learning, we hope that our results can also increase the public’s awareness of the educational significance of their emotional expressions to young children.

More broadly, the ability to use others’ surprise as a proxy signal for a discrepancy between an expected and actual outcome can be characterized as an interesting case of learning from *vicarious prediction error*. In prior work, this term has been used in neuroscience studies that found neural signals that reflect another agent’s expected reward and prediction error (Apps et al., 2015; Burke et al., 2010; Collette et al., 2017; Lockwood et al., 2015; Ruff & Fehr, 2014); the idea is that participants may have “vicariously experienced” someone else’s prediction error by observing the outcomes of another agent’s value-based decision. Here, we are not making claims about infants’ internal experience of the experimenter’s prediction error; unlike prior work, infants in our study could not see the outcome when the experimenter peeked and expressed surprise. These findings should also be distinguished from prior work that focused on how prediction error shapes infants’ subsequent learning or expectations about future outcomes (e.g., Stahl & Feigenson, 2015). While the role of others’ prediction error on infants’ subsequent learning remains an exciting question for future work, the current work focuses on infants’ inferences about an outcome that has already occurred. We demonstrate that when the actual outcome of an event is inaccessible to a learner, the learner can learn vicariously about the hidden outcome by using another agent’s expressions of surprise, an indicator of that agent’s prediction error. Taken together, going beyond past work that operationalize social information as a relatively simple reward-predictive cue (Vélez & Gweon, 2021), future research might further investigate the cognitive and neural mechanisms that underlie our ability to learn vicariously from others’ prediction error, and in particular, how others’ emotional expressions can provide a useful source of information for such learning.

To conclude, the current study demonstrates the effect of others’ surprise on infants’ expectations about event outcomes. For decades, infants’ looking time has been assumed to be longer for unexpected or surprising events; this pattern—a behavioral marker of prediction error—has been an essential tool for developmental scientists to investigate what infants know about the world (Spelke, 2022). Our results, however, show that infants’ inferential abilities in social contexts are powerful enough to modulate this standard pattern, making surprising events “surprisingly unsurprising.”

## ACKNOWLEDGMENTS

Our acknowledgments go to the Palo Alto Museum and Zoo, where our data were collected, and to the participating families and children. We would also like to express our appreciation to the students who coded the data for this study.

## FUNDING INFORMATION

This work is funded by a Jacobs Foundation Research Fellowship and a grant (#2019567) from National Science Foundation (NSF) awarded to Hyowon Gweon, as well as a Discovery Grant from the Natural Sciences and Engineering Research Council of Canada (NSERC) awarded to Yang Wu.

## AUTHOR CONTRIBUTIONS

Y.W.: Conceptualization: Equal; Formal analysis: Equal; Investigation: Supporting; Methodology: Equal; Writing – original draft: Equal; Writing – review & editing: Equal. M.M.: Investigation: Lead; Methodology: Supporting. H.G.: Conceptualization: Equal; Formal analysis: Equal; Funding acquisition: Lead; Investigation: Supporting; Methodology: Equal; Writing – original draft: Equal; Writing – review & editing: Equal.

## DATA AVAILABILITY STATEMENT

All data and analysis code are available at https://osf.io/wdz3k/?view_only=bb3e7f89dc1846ea8f8629fda2ac9580.

## Notes

^1^ In Xu and Garcia’s study (2008), the probabilities of the improbable and probable outcomes were 0.000023 and 0.27, respectively; in our study, they were 0.05 and 0.95, respectively, suggesting that our improbable outcome was less improbable compared to Xu and Garcia (2008). Indeed, a previous study with 8- to 11-month-olds found relatively fragile effects using a single ball sampled from a large population (Yeung et al., 2016). Given that we focus on older infants, we ran a conceptual replication of the original task (Xu & Garcia, 2008) with infants aged 12 to 17 months using a single-ball sample, and found a small-to-medium effect size (Cohen’s *d* = .31) for the difference in looking time between an improbable vs. probable outcome. Please see Supplementary Online Material (SOM) for more details.^2^ While our stimuli were presented live, demo videos for the familiarization and test trials are available at https://osf.io/4s9p2/?view_only=076b902120d54702a27920bd33c60811 for reference.^3^ Model: lme(log(Looking Time) ∼ Probability * Emotion, random = ∼ Probability * Emotion | Subject, method = “REML”).^4^ Preregistrations are available at: https://aspredicted.org/blind.php?x=ky87u8 (Experiment 2a) and https://aspredicted.org/blind.php?x=gu5h23 (Experiment 2b).^5^ Although this analysis was included in Experiment 2b preregistration, Experiment 2a data had already been collected at the time of the preregistration.^6^ While the effect of age was not included in our pre-registered confirmatory or exploratory analyses, we explored age effects in all experiments as suggested by an anonymous reviewer. We used a mixed-effects model: lme(log(Looking Time) ∼ Probability * Emotion * Age, random = ∼ Probability * Emotion | Subject, method = “REML”), where Age is a continuous variable and centered. In Experiment 2a, we found that with age, infants were more likely to show the predicted pattern of response in the Unsurprised trials (*B* = −.27, *β* = −.39, *t*(52) = −2.47, *p* = .017), suggesting that older infants were less likely to be affected by seeing a salient emotional expression (i.e., surprise) early in the experiment than younger infants. No similar age effect was found in Experiment 1, the Surprised trials in Experiment 2a, or Experiment 2b.

## Supplementary Material


