# Supplementary material for: Expecting the Unexpected: Infants Use Others’ Surprise to Revise Their Own Expectations
Source: Open Mind (Camb). 2024 Mar 1;8:67–83. doi: 10.1162/opmi_a_00117 (PMC10898783; doi:10.1162/opmi_a_00117)
Supplement: Supplementary file 1 [file opmi-08-67-s001.pdf]

Supplementary Online Materials for

Expecting the unexpected: Infants use others' surprise to revise their own expectations

1. Manipulation check: Conceptual Replication of Xu & Garcia (2008)

Participants. We estimated the effect size in Xu & Garcia (2008, Experiments 1, 2, 4, and 5; mean Cohen's $d = .55$) to calculate our sample size. The power analysis suggested a sample of $N = 28$ to reach 80% power. Thus, our final sample included 28 infants (mean: 15.5 months, range: 12.4–17.7; 8 girls, 20 boys) recruited from the same local museum as in the main experiments, with full data from 22 infants and partial data from 6 infants. Partial data resulted from the exclusion of 9 trials due to fussiness (4 trials) and experimenter error (5 trials). Another two infants had insufficient data after trial-level exclusion and were excluded from further analysis.

Materials. We used the same materials as in Experiment 1.

Procedure. The procedure was the same as in Experiment 1 except that in the test trials, the experimenter neither revealed the outcome to herself nor reacted to it. Instead, she revealed the outcome to infants immediately after she sampled a ball and put it in the small container. We used the same procedure to code infants' looking time as in Experiments 1 and 2. The primary offline coder judged that the experimenter ended 5 trials prematurely, which were then excluded (categorized as "experimenter error" in data exclusion; see*Participants*). The reliability between the primary and second offline coders was $r = .84$; large discrepancies were resolved by discussion.

Results. As the raw looking time data violated the assumption of normality ($p < 10^{-9}$), we followed the recommendation to log-transform the data before excluding outliers and analysis (Csibra, Hernik, Mascaro, Tatone, & Lengyel, 2016). The data was normally distributed after log transformation ($p = .947$) and all data points were within three deviations of the mean. Infants' tendency to look longer at the improbable outcome than the probable outcome did not reach statistical significance ($t(26) = 1.60$, $p = .121$, 95% CI [-.06, .51], Cohen's $d = .31$; paired t-test).

The effect size is reasonable given prior work. It is smaller than the effect size reported in the original task (Xu & Garcia, 2008), likely because our study involved sampling a *single* ball from a large population, resulting in a relatively less surprising improbable outcome compared to the original study (i.e., the probabilities of the improbable and probable outcomes were 0.05 and 0.95, respective, in our study, and 0.000023 and 0.27, respectively, in the original study). On the other hand, our effect size is larger than those observed in other studies that used single-ball sampling from a large set but focused on the looking times of younger infants (Téglás, Ibanez-Lillo, Costa, & Bonatti, 2015; Yeung, Denison, & Johnson, 2016), suggesting that the ability to reason about single-ball samples improves with age.

Importantly, although this conceptual replication yielded results that did not reach statistical significance, it nonetheless provided reasonable grounds for our main experiments. Our

primary focus is not on the effect of event probability per se, but on whether this baseline effect could be modulated by the experimenter's emotional expression: preserved or amplified when the experimenter was unsurprised, and reduced or even flipped when the experimenter was surprised. Thus, finding a small baseline effect did not raise significant concerns. Our main paper further confirms the modulation effect of emotion on this baseline effect (please see Results and Figure 2 in the main text).

3. Legends for Movies S1 to S5

Movie S1. Demo video for familiarization trials
Movie S2. Demo video for the unsurprised-probable trial
Movie S3. Demo video for the unsurprised-improbable trial
Movie S4. Demo video for the surprised-probable trial
Movie S5. Demo video for the surprised-improbable trial

References

Csibra, G., Hernik, M., Mascaro, O., Tatone, D., & Lengyel, M. (2016). Statistical treatment of looking-time data. *Developmental psychology*, *52*(4), 521.

Téglás, E., Ibanez-Lillo, A., Costa, A., & Bonatti, L. L. (2015). Numerical representations and intuitions of probabilities at 12 months. *Developmental science*, *18*(2), 183–193.

Xu, F., & Garcia, V. (2008). Intuitive statistics by 8-month-old infants. *Proceedings of the National Academy of Sciences*, *105*(13), 5012–5015.

Yeung, H. H., Denison, S., & Johnson, S. P. (2016). Infants' looking to surprising events: When eye-tracking reveals more than looking time. *PloS one*, *11*(12), e0164277.
